# Pressure and stretch differentially affect proliferation of renal proximal tubular cells

**DOI:** 10.14814/phy2.13346

**Published:** 2017-09-14

**Authors:** Diane Felsen, Bianca J. Diaz, Jie Chen, Juana Gonzalez, Marie Louise V. Kristensen, Anja B. Bohn, Brendan T. Roth, Dix P. Poppas, Rikke Nørregaard

**Affiliations:** ^1^ Department of Urology Institute for Pediatric Urology Komansky Center for Children's Health Weill Cornell Medicine New York New York; ^2^ Center for Clinical and Translational Science Rockefeller University New York New York; ^3^ Department of Clinical Medicine Aarhus University Aarhus C. Denmark

**Keywords:** Cell cycle, pressure, proliferation, renal tubules, skp2, stretch

## Abstract

Renal obstruction is frequently found in adults and children. Mechanical stimuli, including pressure and stretch in the obstructed kidney, contribute to damage; animal models of obstruction are characterized by increased cellular proliferation. We were interested in the direct effects of pressure and stretch on renal tubular cell proliferation. Human HKC‐8 or rat NRK‐52E proximal tubule cells were subjected to either pressure [0, 60 or 90 mmHg] or static stretch [0 or 20%] for 24 or 48 h. Cell proliferation was measured by cell counting, cell cycle analyzed by flow cytometry, and PCNA and Skp2 expression were determined by qPCR or western blot. Blood gases were determined in an iSTAT system. Proliferation was also assessed in vivo after 24 h of ureteral obstruction. There was a significant increase in HKC‐8 cell number after 48 h of exposure to either 60 or 90 mmHg pressure. Western blot and qPCR confirmed increased expression of PCNA and Skp2 in pressurized cells. Cell cycle measurements demonstrated an increase in HKC‐8 in S phase. Mechanical stretching increased PCNA protein expression in HKC‐8 cells after 48 h while no effect was observed on Skp2 and cell cycle measurements. Increased PCNA expression was found at 24 h after ureteral obstruction. We demonstrate direct transduction of pressure into a proliferative response in HKC‐8 and NRK‐52E cells, measured by cell number, PCNA and Skp2 expression and increase in cells in S phase, whereas stretch had a less robust effect on proliferation.

## Introduction

Obstruction of the kidney is a frequent finding in adults, resulting from a variety of causes, including calculi, tumors of the kidney or ureter, inflammation, benign prostatic hyperplasia and a host of other metabolic factors (Gulmi and Felsen 2012). Congenital anomalies of the kidney and urinary tract are the most frequently detected abnormalities found during prenatal ultrasound, and can lead to long‐term renal dysfunction in children (Yamaçake and Nguyen 2013). We and others (Miyajima et al. 2000b; Klahr and Morrissey 2002; Mizuguchi et al. 2008; Norregaard et al. 2009; Oestergaard et al. 2014) have used a model of ureteral obstruction in which there is complete unilateral ureteral obstruction (UUO) to study the pathological processes involved in UUO. In acute UUO, there is an immediate marked elevation of ureteral pressure, increasing from 50 to 80 cm H_2_O (Moody et al. 1975). Over time, there is a sustained increase in interstitial pressure in the kidney (Wyker et al. 1981). Long‐term UUO can impart mechanical strain on the kidney, resulting in dilatation and stretch of the collecting system, parenchymal thinning, tubular atrophy, interstitial infiltration and fibrosis, and significant loss in kidney weight and function (Vaughan et al. 2004).

In order to study the effects of obstruction on renal cellular function, investigators have used models of both in vitro pressure and stretch. We developed a simple system to apply pressure directly to cultured cells in vitro. Using this system we found a short‐term induction of inducible nitric oxide synthase, which was mediated through the epidermal growth factor receptor, and subsequent signaling through NF‐*κ*B and STAT‐3 (Broadbelt et al. 2009, 2007). We and others have used in vitro stretch of cells as an in vitro model of UUO (Miyajima et al. 2000a). Such studies have, in renal cells, demonstrated that stretch can increase the pro‐inflammatory enzyme COX‐2 in renal medullary interstitial cells (Carlsen et al. 2015) and upregulate extracellular matrix proteins, including TGF*β* and fibronectin in proximal tubular epithelial cells (Hamzeh et al. 2015) and fibroblasts (El Chaar et al. 2005; Oestergaard et al. 2014).

The obstructed kidney is characterized by changes in cellular proliferation, as measured by proliferating cell nuclear antigen [PCNA] and apoptosis. Truong et al. (1996) demonstrated that there was a rapid rise in proliferation of renal tubular cells within the first 10 days of UUO; this was followed by a decline in tubular proliferation, and an increase in tubular apoptosis. The stimulus for this is not known, but may involve mechanotransduction of the pressure signal. Cell proliferation involves progression of cells through the cell cycle; a complex network of cyclins, cyclin‐dependent kinases (CDK) and CDK inhibitors [CDKI] control this process (Suzuki et al. 2013). P27 is a CDKI which undergoes ubiquitination prior to its degradation, and the F‐box protein Skp2 is a rate‐limiting component of this process (Carrano et al. 1999). Furthermore, deletion of Skp2 in the UUO model ameliorates damage, suggesting an important role for Skp2 in the kidney (Suzuki et al. 2007).

There have been several reports of the effect of pressure and or mechanical stretch on proliferation of various cell types. Vascular smooth muscle cells and bladder smooth muscle cells subjected to a range of pressures in vitro show increased proliferation (Chen et al. 2012; Luo et al. 2010). Gastric epithelial cells under pressure, and pulmonary epithelial cells (Chess et al. 2005) and fibroblasts exposed to stretch also have been shown to exhibit (Nakamizo et al. 2012; Wang et al. 2005) increased proliferation. Interestingly, an in vivo study of the obstructed kidney, specifically examining the urothelium covering the papilla, noted increased proliferation within 2 days of obstruction, which decreased when the obstruction was removed (Girshovich et al. 2012).

Therefore, in this study, we were interested in whether pressure or stretch would affect proliferation of human renal epithelial cells directly. Since we use the rat model of UUO extensively, we were also interested in whether pressure would also affect proliferation of rat epithelial cells. In addition to proliferation [assessed by cell counting and PCNA expression], we examined the effects of pressure and stretch on changes in cell cycle and Skp2 expression. Finally, we examined PCNA expression in vivo at 24 h following ureteral obstruction.

## Materials and Methods

### Application of pressure to cells

HKC‐8 [Human renal epithelial] or NRK52E [normal rat kidney; NRK] cells were grown in a humidified atmosphere of 5% CO_2_‐95% air at 37°C in DMEM with low glucose (Gibco, Thermo Fisher Scientific, NY) supplemented with 10% FBS, penicillin and streptomycin. Cells were suspended in complete medium and cultured in 24‐well plates. When cells reached 70–80% confluence, medium was changed, and in half the wells, medium was replaced with fresh serum‐free medium (DMEM medium supplemented with penicillin and streptomycin only); the other half with fresh complete medium (DMEM supplemented with penicillin and streptomycin as well as 10% FBS).

Pressure was applied to the cell lines through a customized pressure system developed in our laboratory (Broadbelt et al. 2009, 2007). The cells were treated with 0, 60, or 90 mmHg pressure for 24 or 48 h.

### Application of stretch to cells using FlexCell apparatus

The effect of stretch on HKC‐8 and NRK‐52E cells was studied using the FlexCell FX‐5000T^™^ system (Dunn Labortechnik GmbH, Asbach, Germany), which applies stretch to adhesive cell types (Brown 2000). Reaching 100% confluency, HKC‐8 and NRK‐52E cells were subcultured into six‐well collagen‐coated BioFlex plates (Dunn Labortechnik GmbH, Asbach, Germany) at a density of 50% confluency. After culture for 12 h, the medium was changed, and in half the wells, medium was replaced with fresh serum‐free medium, and in the other half with fresh complete medium. Afterward, the cells were exposed to uniform static stretch for 24 and 48 h. To determine the optimal conditions, we applied different amounts of static stretch to the cells and increased the attached cell surface area by 10%, 15%, and 20% using a frequency of 1 Hz (Oestergaard et al. 2014). As a control, non‐stretched cells were used. The complete system was placed in a CO_2_ incubator to maintain the temperature, humidity, and atmosphere during the stretch experiment. In the optimal condition, stretch of 0% (control) and 20% was applied to the HKC‐8 and NRK‐52E cells for 24 or 48 h.

### Medium composition

To determine the effect of pressure on the culture medium, pH, pO_2_, pCO_2_ and HCO_3_ were measured using an Abbott i‐STAT blood gas analyzer.

### Cell number

Cell number was determined using the fluorometric CyQUANT assay (Thermo Fisher Scientific, Cambridge, MA) according to manufacturer's protocol.

### Flow cytometry

Flow cytometry was used to determine distribution of cells in the cell cycle. For the pressure measurements, cells were incubated as above. They were then permeabilized and stained with Propidium Iodide, using the BD Cycletest Kit. Samples were analyzed using an LSR‐II flow cytometer.

For the FlexCell experiments, cells were washed twice in PBS containing 1 mM EDTA to minimize clumping. After centrifugation (500*g*, 5 min), the supernatant was discarded and the cells were fixed in 1 mL cold 70% ethanol for 30 min on ice. The cells were washed twice with PBS, centrifuged at 350*g* for 5 min and the supernatant was discarded before 500 *μ*L FxCycle^™^ PI/RNase Staining Solution (Life Technologies, Eugene, Oregon) was added. After 15 min of incubation cells were analyzed on a NovoCyte flow cytometer (ACEA Biosciences. Inc., San Diego, CA). The cell cycle was calculated with the “Watson pragmatic model.”

### qPCR

Total RNA was isolated from the cells using TRIzol reagent following the manufacturer's instructions (Thermo Fisher Scientific). RNA was quantitated by spectrophotometry and stored at −80°C. cDNA was synthesized from 0.5 *μ*g RNA with the AffinityScript QPCR cDNA synthesis kit (Thermo Fisher Scientific). For QPCR, 100 ng cDNA served as the template for PCR amplification using the Brilliant SYBR^®^ Green QPCR Master Mix according to the manufacturer's instructions (Life Technologies). PCNA and Skp2 mRNA levels were validated by QPCR (Proliferating Cell Nuclear Antigen (PCNA): forward: 5′ TGG AGA ACT TGG AAA TGG AAA ‘3; reverse: 5′ GAA CTG GTT CAT TCA TCT CTA TGG ‘3 and Skp2: forward: 5′ TCA GGA ATT TTT CCA GCT CAA'3; reverse: 5′ CTG GCA CGA TTC CAA AAA CT'3), with *β*‐actin (forward: 5′ CTG ACA GGA TGC AGA AGG 3′; reverse: 5′ GAG TAC TTG CGC TCA GGA‐3 3′) as the control gene.

### Western blot analysis

Cells were collected and lysed using the M‐PER Mammalian Protein Extraction Reagent (Thermo Scientific, Vedbaek, Denmark). Cell suspensions were centrifuged at 14,000*g* at room temperature for 10 min. Gel samples were prepared from supernatants mixed with Laemmli sample buffer containing 2% SDS. The Pierce BCA Protein Assay Kit (Roche, Hvidovre, Denmark) was used to determine the total protein concentration of homogenates. Proteins were separated on a 12% Criterion TGX Precast Gel (Bio‐Rad Laboratories, Copenhagen, Denmark) and transferred to a Hybond ECL nitrocellulose membrane (GE Healthcare, Hatfield, UK). The membrane was then blocked in 5% non‐fat dry milk in PBS‐T (80 mmol/L Na_2_HPO_4_, 20 mmol/L NaH_2_PO_4_, 100 mmol/L NaCl, 0.1 Tween 20, pH 7.4), washed in PBS‐T, and incubated with primary antibodies overnight at 4°C. Primary antibodies are PCNA (#PC10, Abcam) and SKP2 (#sc7164, Santa Cruz). Subsequently, the membrane was incubated with a HRP‐conjugated secondary antibody at room temperature for 1 h. Antigen‐antibody reactions were visualized using an enhanced chemiluminescence system (Amersham ECL Plus, GE Healthcare). All western blots were normalized to total protein, measured by the Stain‐Free technology (Gürtler et al. 2013) *β*‐actin was used as a loading control.

### In Vivo ureteral obstruction

Sprague‐Dawley rats (*n* = 6) underwent left ureteral ligation at the end of the lower ureter, just above the ureterovesical junction with 4–0 silk suture (Broadbelt et al. 2009). A midline abdominal incision was made under sterile conditions. Animals were anesthetized with ketamine‐xylazine cocktail. Obstructed and contralateral kidneys were harvested at 24 h. Animal procedures performed in accordance with experimental protocols approved by the Institutional Animal Care and Use Committee at Weill Cornell Medicine according to the NIH Guidelines for the Care and Use of Laboratory Animals. All procedures conformed to Tissues were paraffin embedded and PCNA staining carried out as previously described (Miyajima et al. 2000b; Mizuguchi et al. 2008).

### Statistical analysis

Values are expressed as the mean ± SEM. Data was analyzed by one way ANOVA and student's *t*‐test with Bonferroni adjustment using GraphPad Prism Software. A probability level of 0.05 (*P* ≤ 0.05) was considered significant.

## Results

### Effect of pressure on the milieu of the cells

We were interested in whether pressure or stretch would induce proliferation in renal epithelial cells. Prior to starting these experiments, we wanted to determine if pressurization was affecting the milieu of the cells. Therefore, we incubated cells for 24 h in serum‐free medium [DMEM only] at pressure of 60 mmHg, in our previously described system (Broadbelt et al. 2009, 2007). We measured pH, pCO_2_, pO_2_ and HCO_3_ at 24 h. Results, shown in Table 1, indicate that pressure had no effect on pH, pO_2_ or HCO_3_. There was a slight, but significant effect on pCO2, which increased from 28.9 ± 0.5 to 31.9 ± 0.6. Although statistically significant, this change was not considered to have a physiologic impact on the experiments.

**Table 1:** Effect of pressure incubation on medium composition

	pH	pCO_2_	pO_2_	HCO_3_
No pressure	7.5 ± 0.01	28.9 ± 0.5	146 ± 2.6	25.4 ± 0.3
60 mmHg	7.5 ± 0.02	31.9 ± 0.6^a^	152.7 ± 3.1	25.9 ± 0.5

Results are means of 6 experiments.

a *P* < 0.05 compared to no pressure.

### Effect of pressure and stretch on cell number

**Figure 1:**
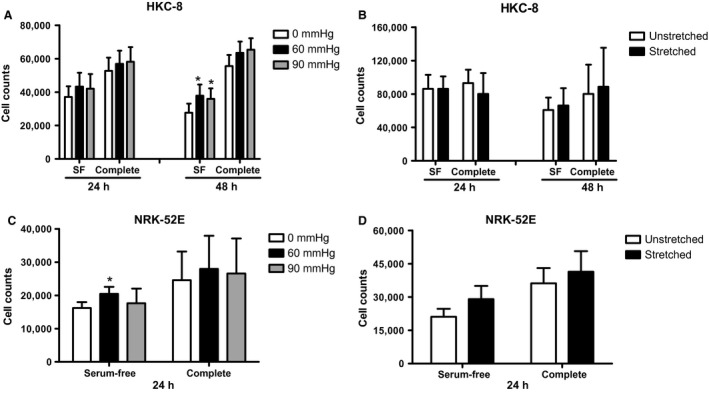
Effect of pressure and stretch on cell number in renal tubular cells. (A) HKC‐8 cells were incubated with either SF or complete medium, for 24 or 48 h under pressure. (B) HKC‐8 cells were incubated with either SF or complete medium, for 24 or 48 h under stretch. (C) NRK‐52E cells were incubated with either serum‐free or complete medium, for 24 under pressure. (D) NRK‐52E cells were incubated with either serum‐free or complete medium, for 24 h under stretch. Data represents mean ± SEM. *P < 0.05 compared to zero pressure. *N* = 3, with triplicate replications in each experiments. SF, serum‐free.

We then examined the effect of pressure and stretch on cell number. We utilized HKC‐8, a human renal proximal tubular cell line. Cells were incubated in either complete or serum‐free medium for 24 or 48 h at 0, 60 and 90 mmHg. Application of both 60 and 90 mmHg pressure to cells in serum‐free medium significantly increased cell number at 48 h (Fig. 1A). There was a significant increase from 27656 ± 5436 cells/well at zero pressure to 37809 ± 6690 cells/well at 60 mmHg pressure, a 39.2 ± 5.1% increase. There was no change in cell number with both 60 and 90 mmHg pressure in complete medium (Fig. 1A).

We also examined the effect of stretch on cell number. Cells underwent a static stretch of 20% in either complete or serum‐free medium for 24 or 48 h. At both 24 and 48 h, there was no significant effect of stretch on cell number in either serum‐free or complete medium (Fig. 1B).

Since we have used the rat model of UUO in many studies, we were interested in determining if rat renal epithelial cells responded similarly to pressure and stretch. We used the NRK‐52E cell line for these experiments. In serum‐free medium, 60 mmHg pressure increased cell number from 16224 ± 1726 cells per well to 20488 ± 2081 cells/well [*P* < 0.05], a 26.8 ± 2.6% increase (Fig. 1C). In complete medium, no change in cell number was observed. There was no significant increase in cell number when the cells were stretched for 24 h (Fig. 1D).

### Effect of pressure and stretch on cells in cell cycle

We were then interested in determining if pressure and stretch could drive cells from the G1 phase of the cell cycle into the S or G2 phase. Therefore, we incubated cells for 24 or 48 h as above. Cells were then analyzed by flow cytometry using the Watson pragmatic model.

**Figure 2:**
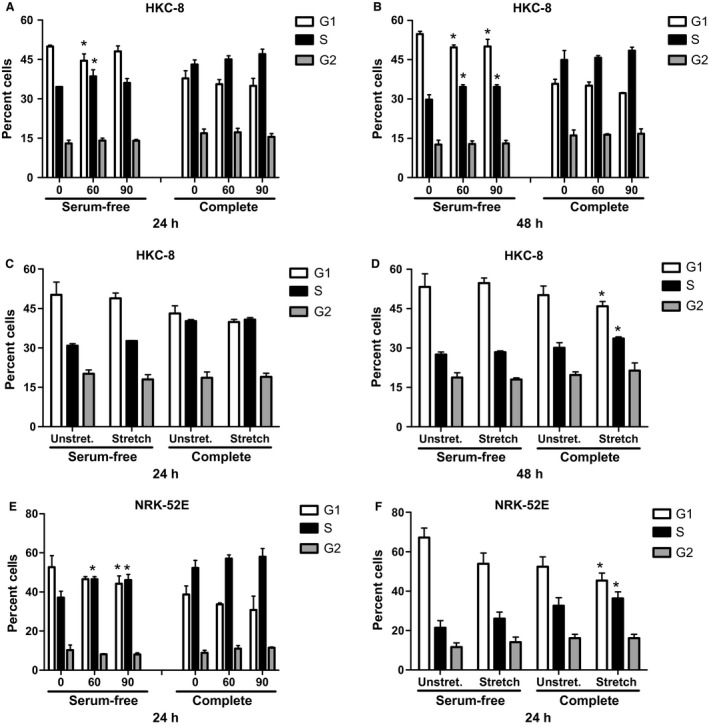
Effect of pressure and stretch on cell cycle in tubular cells. HKC‐8 or NRK‐52 E cells were pressurized or stretched and subject to flow cytometric analysis. HKC‐8 cells pressurized for 24 (A) or 48 (B) h. HKC‐8 cells stretched for 24 (C) or 48 (D) h. NRK 52 E cells pressurized (E) or stretched (F) for 24 h. Data represents mean ± SEM. *P < 0.05 compared to zero pressure or unstretched cells. *N* = 3, with triplicate replications in each experiments.

A pressure of 60 mmHg significantly increased entry into S phase in serum‐free medium at both 24 and 48 h in the HKC‐8 cells (Fig. 2A–B). At 24 h, the percent of cells in S phase at zero pressure was 34.5 ± 0.1, which was increased to 38.6 ± 1.4% at 60 mmHg pressure. There was a corresponding decrease in the % of cells in G1 from 49.9 ± 0.3% to 44.5 ± 1.5% (Table 2). At 90 mmHg pressure, there was a significant effect only at 48 h (Fig. 2B). In complete medium at both 24 and 48 h, there was no effect of pressure (Fig. 2A–B). Stretch significantly increased the percent of HKC‐8 cells in S phase in complete medium at 48 h with a corresponding decrease in the G1 phase (Fig. 2D). The percent of non‐stretched control cells in the S phase was 30.2 ± 1.1%, which was increased to 33.7 ± 0.4% at 48 h stretch (Table 3). There was no effect of stretch on the cell cycle status at 24 h (Fig. 2C).

**Table 2:** Phases of the cell cycle in pressurized HKC‐8 and NRK‐52E cells

Cell cycle phase	Serum‐free HKC‐8 cells			Complete HKC‐8 cells		
	0 mmHg (%)	60 mmHg (%)	90 mmHg (%)	0 mmHg (%)	60 mmHg (%)	90 mmHg (%)
24 h						
G_0_/G_1_	49.9 ± 0.3	44.5 ± 1.5^a^	48.1 ± 1.2	37.8 ± 1.7	35.6 ± 1.0	34.9 ± 1.7
S	34.5 ± 0.1	38.6 ± 1.4^a^	36.0 ± 1.0	43.1 ± 1.0	45.0 ± 0.8	47.0 ± 1.1
G_2_/M	13.0 ± 0.7	14.1 ± 0.5	14.0 ± 0.3	16.9 ± 0.9	17.2 ± 0.9	15.5 ± 0.7
48 h						
G_0_/G_1_	54.8 ± 0.6	49.7 ± 0.5^a^	50.0 ± 1.6^a^	35.8 ± 1.0	35.1 ± 0.8	32.3 ± 0.1
S	29.7 ± 1.1	34.6 ± 0.5^a^	34.6 ± 0.5^a^	44.9 ± 2.1	45.7 ± 0.5	48.4 ± 0.8
G_2_/M	12.6 ± 1.0	12.8 ± 0.7	13.0 ± 0.7	16.1 ± 1.2	16.4 ± 0.2	16.7 ± 1.1

Cell cycle phase	Serum‐free NRK‐52E cells			Complete NRK‐52E cells		
	0 mmHg (%)	60 mmHg (%)	90 mmHg (%)	0 mmHg (%)	60 mmHg (%)	90 mmHg (%)
24 h						
G_0_/G_1_	52.7 ± 3.4	46.6 ± 0.7	44.2 ± 2.3^a^	38.7 ± 2.5	33.7 ± 0.4	30.8 ± 4.1
S	37.1 ± 1.9	46.6 ± 0.7^a^	46.0 ± 1.7^a^	52.3 ± 2.2	57.0 ± 1.1	58.0 ± 2.4
G_2_/M	10.3 ± 1.5	8.2 ± 0.1	8.0 ± 0.5	8.8 ± 0.8	11.0 ± 0.9	11.5 ± 0.2

The percentage of cells in each phase of the cell cycle is shown in pressurized cells cultured in either serum‐free medium (DMEM only) or in complete medium (DMEM + 10% FBS) at each time point. Data represents mean ± SEM.

a *P* < 0.05 compared to zero pressure. *N* = 3, with triplicate replications in each experiments.

**Table 3:** Phases of the cell cycle in stretched HKC‐8 and NRK‐52E cells

Cell cycle phase	Serum‐free HKC‐8 cells		Complete HKC‐8 cells	
	Unstretched (%)	Stretched (%)	Unstretched (%)	Stretched (%)
24 h				
G_0_/G_1_	50.2 ± 2.8	48.8 ± 1.2	43.1 ± 1.7	39.9 ± 0.5
S	29.7 ± 0.5	32.6 ± 0.9	40.2 ± 0.4	40.7 ± 0.5
G_2_/M	20.1 ± 0.9	18.0 ± 1.0	18.6 ± 1.3	19.0 ± 0.8
48 h				
G_0_/G_1_	53.3 ± 2.8	54.8 ± 1.1	50.2 ± 2.0	45.9 ± 1.0^a^
S	27.6 ± 0.5	28.4 ± 0.3	30.2 ± 1.1	33.7 ± 0.4^a^
G_2_/M	18.8 ± 1.1	18.0 ± 0.4	19.8 ± 0.7	21.4 ± 1.7

Cell cycle phase	Serum‐free NRK‐52E cells		Complete NRK‐52E cells	
	Unstretched (%)	Stretched (%)	Unstretched (%)	Stretched (%)
24 h				
G_0_/G_1_	67.2 ± 2.8	53.9 ± 3.2	52.5 ± 2.8	45.4 ± 2.2^a^
S	21.4 ± 2.1	26.0 ± 1.9	33.6 ± 2.4	36.3 ± 1.9^a^
G_2_/M	11.5 ± 1.3	14.1 ± 1.5	16.1 ± 1.2	16.1 ± 1.1

The percentage of cells in each phase of the cell cycle is shown in pressurized cells cultured in either serum‐free medium (DMEM only) or in complete medium (DMEM + 10% FBS) at each time point. Data represents mean ± SEM.

a *P* < 0.05 compared to unstretched cells. *N* = 3, with triplicate replications in each experiments.

Pressure had similar effects in the NRK‐52E cells. Incubation of NRK‐52E cells for 24 h in serum‐free medium in the presence of 60 or 90 mmHg pressure significantly increased the percent of cells in S phase (Fig. 2E, Table 2). In addition, 24 h stretch increased the percent of NRK‐52E cells in the S phase in complete medium (Fig. 2F, Table 3).

### Effect of pressure and stretch on PCNA and Skp2 expression

**Figure 3:**
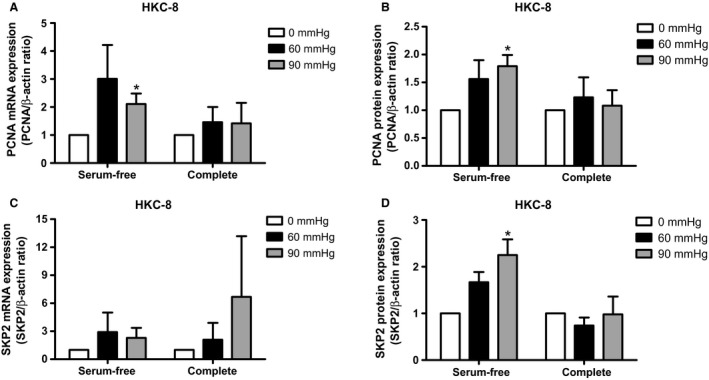
PCNA and SKP2 expression in pressurized cells. HKC‐8 cells were pressurized and qPCR and western blot were carried out. PCNA mRNA (A) and protein (B) expression in HKC‐8 cells pressurized for 24 h. Skp2 mRNA (C) and protein (D) expression in HKC‐8 cells pressurized for 24 h. Data represents mean ± SEM. *P < 0.05 compared to zero pressure. *N* = 3, with triplicate replications in each experiments.

To determine if the increase in cell number reflected increased cell proliferation, we examined cells for expression of PCNA and Skp2, by QPCR and western blot analysis. HKC‐8 cells incubated for 24 h under 60 and 90 mmHg pressure exhibited increased PCNA mRNA (Fig. 3A) and protein expression (Fig. 3B) in serum‐free medium. In addition, Skp2 protein expression was increased in starved HKC‐8 cells at both 60 and 90 mmHg at 24 h (Fig. 3D) but no change was observed at the mRNA level (Fig. 3C). No change in PCNA and Skp2 mRNA and protein expression was observed in complete medium (Fig. 3A–D)

**Figure 4:**
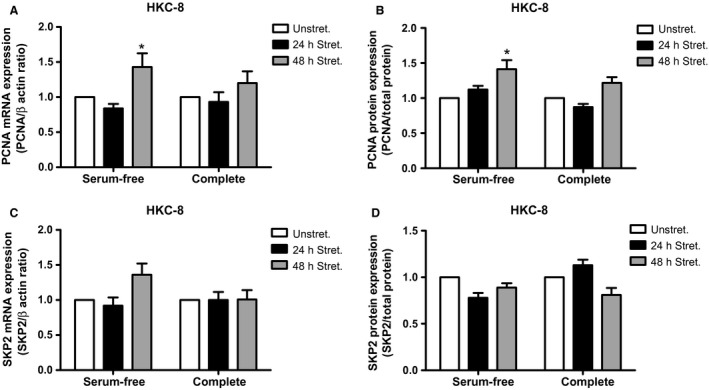
PCNA and SKP2 expression in stretched cells. HKC‐8 or NRK‐52 E cells were stretched and qPCR and western blot were carried out. PCNA mRNA (A) and protein (B) expression in stretched HKC‐8 cells. Skp2 mRNA (C) and protein (D) expression in stretched HKC‐8 cells. Data represents mean ± SEM. *P < 0.05 compared to unstretched cells. *N* = 3, with triplicate replications in each experiments.

In order to determine if stretch similarly could affect the expression of PCNA and Skp2 in the HKC‐8 cells, we exposed the cells to static stretch for 24 and 48 h. There was no effect of stretch on PCNA mRNA and protein expression after 24 h (Fig. 4A–B). However, after 48 h stretch we observed increased PCNA mRNA and protein expression in starved HKC‐8 cells (Fig. 4A–B). We observed no change in PCNA and Skp2 mRNA or protein expression after 24 or 48 h stretch in complete medium (Fig. 4A–D).

There was no significant effect of either pressure or stretch treatment for 24 h on PCNA or Skp2 protein expression in NRK 52E cells (data not shown).

### Effect of epidermal growth factor receptor blocker on PCNA expression in response to pressure

**Figure 5:**
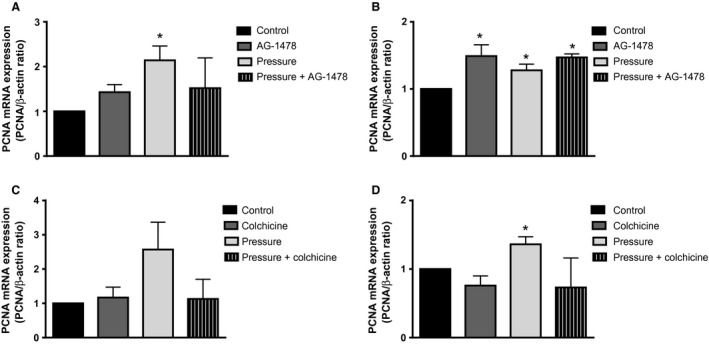
Effect of epidermal growth factor receptor inhibitor or colchicine in pressurized cells. HKC‐8 cells were pressurized (60 mmHg) for 24 h and qPCR and western blot were carried out. PCNA mRNA (A,C) and protein (B,D) expression are shown. Cells were treated with either AG‐1478 [15 *μ*mol/L; A, B] or colchicine [5 nmol/L, C, D]. Data represents mean ± SEM. *P < 0.05 compared to zero pressure. *N* = 3, with triplicate replications in each experiments.

We had previously shown that in HKC‐8 cells, pressure promoted EGF shedding and activation of the EGF receptor (Broadbelt et al. 2009). We were therefore interested in the effects of EGFR blockade on the PCNA response to 60 mmHg pressure for 24 h. We incubated HKC‐8 cells in serum‐free medium in the presence of AG‐1478 (15 *μ*mol/L), an EGFR blocker we had previously utilized (Broadbelt et al. 2009). At the mRNA level, AG‐1478 had no effect on PCNA expression (Fig. 5A). When we examined PCNA protein, we found that both AG‐1478 and pressure significantly increased PCNA expression, indicating that AG‐1478 was unable to block the effect of pressure on PCNA (Fig. 5B).

### Effect of Colchicine on PCNA in response to pressure

Colchicine is an antimitotic, antiproliferative drug which binds to tubulin, and is used in the treatment of gout (Molad 2002). We were interested in its effects on the response of PCNA to 60 mmHg pressure for 24 h. We examined both PCNA mRNA and protein expression (Fig. 5C and 5D). Pressure increased PCNA protein expression; there was a trend toward decreased PCNA expression, but this did not reach statistical significance. Cell number was increased in response to pressure (27440 ± 5722 cells, compared with 17724 ± 2327). In the presence of colchicine and pressure, cell number was significantly reduced to 8577 ± 190 cells (*P* < 0.05).

### PCNA expression in epithelial cells in response to UUO

**Figure 6:**
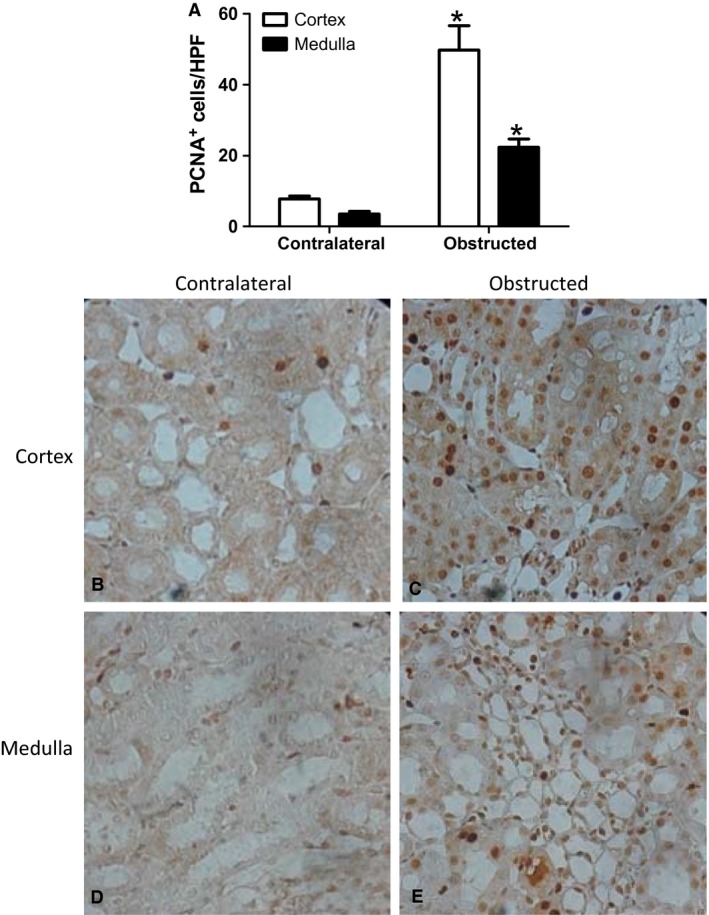
PCNA expression in the obstructed kidney. Rats were subjected to unilateral ureteral obstruction for 24 h. Kidneys were, paraffin embedded and sectioned. PCNA was detected by immunohistochemistry. (A) Graphical representation of counts (B–E): PCNA immunohistochemistry in Kidney Cortex (B–C) and medulla (D–E) after 24 h of UUO. Data represents mean ± SEM. *P < 0.05 compared to obstructed kidney. *N* = 6 rats.

In vitro, we were able to demonstrate an effect of pressure on PCNA expression within 24 h of exposure. We were interested to determine if PCNA expression would also be found in the obstructed kidney at 24 h. Therefore, we obstructed rat kidneys and examined PCNA expression by immunohistochemistry (Fig. 6). There was a significant increase in PCNA in both renal cortex and medulla of the obstructed kidney as compared to the contralateral unobstructed renal cortex (Fig. 6A graphical representation; Fig. 6B–E, immunohistochemistry).

## Discussion

Ureteral obstruction, resulting from conditions including congenital, neoplastic, inflammatory, metabolic and others (Gulmi and Felsen 2012; Vaughan et al. 2004; Yamaçake and Nguyen 2013), leads to increased pressure in the kidney. This has been documented through pressure measurements in the ureter (Moody et al. 1975), through stop‐flow measurements (Arendshorst et al. 1974; Vaughan et al. 2004), and through recording interstitial pressure in the obstructed kidney (Wyker et al. 1981). Increased pressure in the kidney leads to at least two major effects on renal tubular epithelial cells: pure hydrostatic pressure and stretch, but separating these effects in vivo is difficult. To model the obstructed kidney in vitro, we and others used the FlexCell system (Docherty et al. 2006; Oestergaard et al. 2014). In that system, the cells are subject to stretch, which was used in an attempt to mimic stretch which likely occurs in the obstructed kidney. However, since increased pressure is the hallmark of UUO, we developed a system to apply pressure directly to cells.

In this study, we directly compared the effect of pressure with that of stretch in order to investigate whether they influence proliferation in the same way. Our data demonstrated that incubating renal tubular epithelial cells under pressure results in significant proliferation, with a higher percentage of cells found in the S phase of the cell cycle, whereas stretch had a less robust effect on proliferation. This suggests that the increased proliferation of cells found in the in vivo obstructed kidney, may be a more influenced by the increased pressure associated with UUO, than by tubular stretch.

Increased pressure is a pathologic finding in a number of organs including heart and vasculature, lung, liver, gut and bladder. In gastric epithelial cells, pressure has been shown to increase incorporation of BrdU, a measure of proliferation; this was accompanied by an activation of ERK and MEK‐1 pathways (Nakamizo et al. 2012).

In this study, pressure increases the % of cells in the S‐phase as compared to the G1 phase, as assessed by flow cytometry. Similar results have been found in both vascular and bladder smooth muscle cells. Proliferation in vascular smooth muscle cells, assessed by MTT incorporation, was shown to increase with pressure. There was an increase in the proliferation index [cells in S and G2 phase] as compared to cells without pressure (Luo et al. 2010). Increases in proliferation index were also found when bladder smooth muscle cells were subject to pressure (Chen et al. 2012).

Another important component of cell cycle progression is Skp2. Skp2 is an F‐box protein component of the Skp/Cullin/F‐box (SCF)‐type E3 ubiquitin ligase (Suzuki et al. 2013). This ligase is involved in the degradation of the CDKIs p21 and p27 (Bornstein et al. 2003; Carrano et al. 1999). Specifically, it has been shown that Skp2 binds phosphorylated p27 and that ubiquitination of p27 can occur only when Skp2 is bound. Conversely, p27 is stabilized when a dominant negative form of Skp2 replaces wild‐type Skp2 (Suzuki et al. 2013); overexpression of p27 itself inhibits cell cycles progression (Polyak et al. 1994). In pressurized bladder smooth muscle cells, Skp2 is upregulated along with increases in proliferation (Chen et al. 2014); this confirms the results of the present experiments using renal proximal tubules, demonstrating that pressure is a pro‐proliferative signal in a variety of cell types.

We had previously shown that pressure activates the EGF receptor (Broadbelt et al. 2009). In that study we demonstrated that pressure activated inducible nitric oxide within 2 h, which was blocked by incubation of cells with two different EGFR inhibitors. We had anticipated that use of AG‐1478, an EGFR inhibitor would block the effects of pressure on PCNA expression, but this was not found. It is possible that the signaling for effects on PCNA are through a different mechanism, which remains to be determined.

Colchicine is known to target microtubules to exert its antimitotic effects (Molad 2002; Zhou and Giannakakou 2005). In this study, we found that, in response to pressure, increases in both PCNA expression and cell number were blocked by colchicine. In a recent study, colchicine was found to decrease fibrosis in the UUO model (Itano et al. 2017). It was also shown to decrease fibroblast migration in vitro. Tubular cell proliferation was not reported in that study; however, if colchicine blocks tubular cell proliferation in vivo, this could have a negative long‐term effect on the preservation of kidney mass.

In a recent study of the obstructed kidney, Girshovich et al. (2012) examined the effect of obstruction on the phenotype of urothelial cells located at the corticomedullary junction. They noted that there was an increase in proliferation of this population of epithelial cells by 1 day following obstruction. When the obstruction was removed, proliferation was inhibited. This suggests a direct relationship between pressure and proliferation. In this study, we noted a significant increase in proliferation in both cortex and medulla at 24 h after obstruction. Proliferation was not limited to the corticomedullary junction, but was found distributed throughout the kidney. In the study cited above (Girshovich et al. 2012), continued obstruction resulted in a change in the phenotype of these cells, to more closely resemble bladder urothelium, including expression of uroplakins, normally associated exclusively with bladder urothelium. These changes are accompanied by changes in the FGF pathway. It would be interesting to see if these changes can be recapitulated in the in vitro pressure system.

Our study demonstrated less significant effects of stretch on proliferation. Consistent with our study, in mesenchymal stem cells it has been demonstrated that pressure increases proliferation more profoundly than does stretch (Maul et al. 2011), indicating that cells might be more sensitive to pressure compared to stretch. Our results show that when grown in the presence of serum, stretching cells increases the % of cells in the S‐phase as compared to the G1 phase. In contrast, PCNA expression did only increase in stretched cells incubated in starvation medium for 48 h, and cell number as well as Skp2 expression did not change at all in stretched cells compared to control cells grown under identical, but non‐stretched conditions. The effect of mechanical stretch on proliferation has been examined in other cell types. Studies have demonstrated that cyclic stretch increased proliferation in human pulmonary epithelial cells (Chess et al. 2000), 3Y1 rat fibroblasts (Wang et al. 2005) and human mesangial cells (Riser et al. 2000) whereas decreased proliferation was observed in stretched podocytes from mice (Petermann et al. 2002). In the present study, we used uniform static stretch in order to mimic the obstruction condition wherein urine is continuously accumulating in the renal tubules because of the obstruction. Under these circumstances, the proximal tubule cells will experience a more predominately static tensile stretch. This suggests that mechanical stretch can have varying effects on proliferation depending on species and cell types. In addition, the selective responses to different types of stretch might also play a role in proliferation.

PCNA plays a central role in faithful replication of DNA; it is a master coordinator of replication fork processes, including both protein‐protein and protein‐DNA interactions (Mailand et al. 2013). Increased PCNA expression was shown in hepatic stellate cells under pressure (Wu et al. 2010) and in the present study. In the obstructed kidney, increased proliferation in both the tubular and interstitial compartments of the kidney has been demonstrated. Truong et al. (1996) described a rapid rise in renal tubular proliferation in UUO, with a slower but more prolonged rise in interstitial cell proliferation. They suggested that interstitial cell proliferation was associated with renal damage. In studies in UUO, we have shown that either using 1D11, a TGF‐*β* inhibitor, or SS‐31, a mitochondrial – targeted antioxidant, inhibition of fibrosis and decreased renal damage are associated with increased proliferation of renal tubular cells (Miyajima et al. 2000b; Mizuguchi et al. 2008). In contrast, mice in which p27 has been deleted, and whose kidneys show decreases in tubular proliferation, exhibit decreased renal damage in response to UUO, including decreased renal tubular dilatation and interstitial fibrosis (Suzuki et al. 2007). The gene for Skp2 is a global knockout present from birth in mice, whereas our drug treatments were started in mature rats; these differences may contribute to the divergent results of these studies. From our results, the effect of pressure on cellular proliferation may represent an attempt by the kidney to preserve renal cells in the presence of obstruction. Several signaling pathways have been implicated in studies on other organs, and such pathways, or others specific to the obstructed kidney, may be important targets of therapeutic intervention.

## Conflict of Interest

None declared
